# Supplementary material for: Pan-cancer molecular subtypes revealed by mass-spectrometry-based proteomic characterization of more than 500 human cancers
Source: Nat Commun. 2019 Dec 12;10:5679. doi: 10.1038/s41467-019-13528-0 (PMC6908580; doi:10.1038/s41467-019-13528-0)
Supplement: Supplementary file 2 — Supplementary Information [file 41467_2019_13528_MOESM2_ESM.pdf]

Supplementary Information

Chen et al. “Pan-cancer molecular subtypes revealed by mass-spectrometry-based proteomic characterization of more than 500 human cancers”

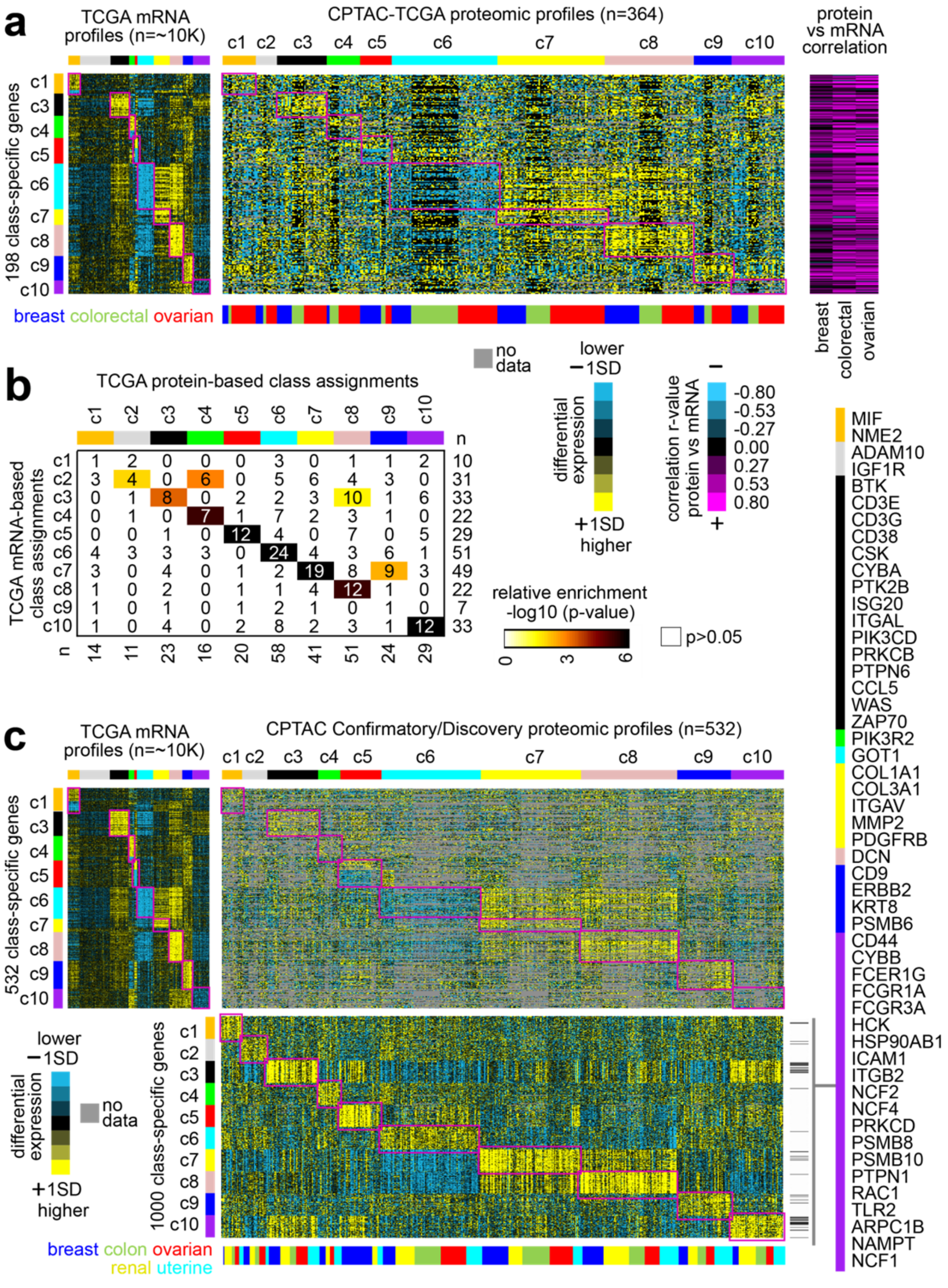
a
TCGA mRNA profiles (n=~10K)
CPTAC-TCGA proteomic profiles (n=364)
protein vs mRNA correlation
c1 c2 c3 c4 c5 c6 c7 c8 c9 c10
198 class-specific genes
c1 c3 c4 c5 c6 c7 c8 c9 c10
breast colorectal ovarian
breast
colorectal
ovarian
no data
lower −1SD
differential expression
+1SD higher
correlation r-value protein vs mRNA
−
-0.80
-0.53
-0.27
0.00
0.27
0.53
0.80
+
b
TCGA protein-based class assignments
TCGA mRNA-based class assignments
c1 c2 c3 c4 c5 c6 c7 c8 c9 c10 n
c1 1 2 0 0 0 3 0 1 1 2 10
c2 3 4 0 6 0 5 6 4 3 0 31
c3 0 1 8 0 2 2 3 10 1 6 33
c4 0 1 0 7 1 7 2 3 1 0 22
c5 0 0 1 0 12 4 0 7 0 5 29
c6 4 3 3 3 0 24 4 3 6 1 51
c7 3 0 4 0 1 2 19 8 9 3 49
c8 1 0 2 0 1 1 4 12 1 0 22
c9 1 0 1 0 1 2 1 0 1 0 7
c10 1 0 4 0 2 8 2 3 1 12 33
n 14 11 23 16 20 58 41 51 24 29
relative enrichment -log10 (p-value)
0 3 6
p>0.05
c
TCGA mRNA profiles (n=~10K)
CPTAC Confirmatory/Discovery proteomic profiles (n=532)
c1 c2 c3 c4 c5 c6 c7 c8 c9 c10
532 class-specific genes
c1 c3 c4 c5 c6 c7 c8 c9 c10
lower −1SD
differential expression
+1SD higher
no data
1000 class-specific genes
c1 c2 c3 c4 c5 c6 c7 c8 c9 c10
breast colon ovarian
renal uterine
MIF
NME2
ADAM10
IGF1R
BTK
CD3E
CD3G
CD38
CSK
CYBA
PTK2B
ISG20
ITGAL
PIK3CD
PRKCB
PTPN6
CCL5
WAS
ZAP70
PIK3R2
GOT1
COL1A1
COL3A1
ITGAV
MMP2
PDGFRB
DCN
CD9
ERBB2
KRT8
PSMB6
CD44
CYBB
FCER1G
FCGR1A
FCGR3A
HCK
HSP90AB1
ICAM1
ITGB2
NCF2
NCF4
PRKCD
PSMB8
PSMB10
PTPN1
RAC1
TLR2
ARPC1B
NAMPT
NCF1

Supplementary Figure 1, related to Figure 1. Pan-cancer molecular classes as defined by transcriptomics are reflected in the cancer proteome. (a) Mass-spectrometry-based proteomic profiles, from the Clinical Proteomic Tumor Analysis Consortium (CPTAC), of 364 cancer cases in The Cancer Genome Atlas (TCGA), were classified according to TCGA mRNA-based pan-cancer molecular class from ref[1]. Expression patterns for a set of 198 mRNAs distinguishing between the ten mRNA-based molecular classes (based on available protein data on the original set of 854 genes, taken from the set of 5863 proteins represented in all three cancer types) are shown for both TCGA "pan32" mRNA (transcriptomes of 32 cancer types and 10224 cases) and CPTAC-TCGA proteomic datasets. For each dataset, expression values are normalized within each main cancer type (SD, standard deviation from the median). Protein patterns in the CPTAC-TCGA sample profiles sharing similarity with mRNA class-specific signature pattern are highlighted. On the right are represented Pearson's correlations between protein and mRNA expression for the cases shared between TCGA and CPTAC. Within each of the three cancer types, correlations between mRNA and protein expression across tumors were generally positive for the 198 features, though with correlation r-values much less than 1 (average Pearson's r-value for colorectal dataset: 0.27; breast: 0.41; ovarian: 0.43), indicative of much of the variation represented by the protein data not being fully captured on the basis of mRNA data. **(b)** For TCGA cases represented in both mRNA and CPTAC proteomic datasets (n=287), significances of overlap between the class assignments made based on mRNA data (rows), with proteomic-based class assignments (columns). P-values by one-sided Fisher's exact test. The statistically significant overlapping assignments being observed here for eight of the ten classes is another indication of transcriptomic patterns being reflected in the proteomic datasets. **(c)** An independent set of mass-spectrometry-based proteomic profiles from CPTAC, of 532 cancer cases not represented in TCGA, were classified according to TCGA mRNA-based pan-cancer molecular class from ref[1]. Expression patterns for a set of 532 mRNAs distinguishing between the ten mRNA-based molecular classes (based on available protein data on the original set of 854 genes, taken from the set of 12247 proteins found for any cancer type) are shown for both TCGA "pan32" mRNA and CPTAC "Confirmation/Discovery" proteomic datasets. For each dataset, expression values are normalized within each main cancer type (SD, standard deviation from the median). The bottom heat map shows differential expression patterns for a set of 1000 proteins in CPTAC Confirmation/Discovery found to best distinguish between the ten classes (see Methods). Proteins highlighted from the bottom heat map have GO[2] annotation "cell surface receptor signaling pathway" and an association in DrugBank[3] database. Large numbers of proteins differentially over-expressed within each of the ten pan-cancer classes can be identified here, whereby differential patterns involving the "c3" and "c10" immune-related classes[1] are found overall to be similar to each other. Particularly in comparison to the epithelial-associated "c6" class, the two stromal-related classes, "c7" and "c8" also appear broadly similar to each other, although proteins with stronger differential patterns can be associated with one class over the other. Some CPTAC cases apparently manifesting a signature associated with the previously identified neuroendocrine-associated "c4" class[1].

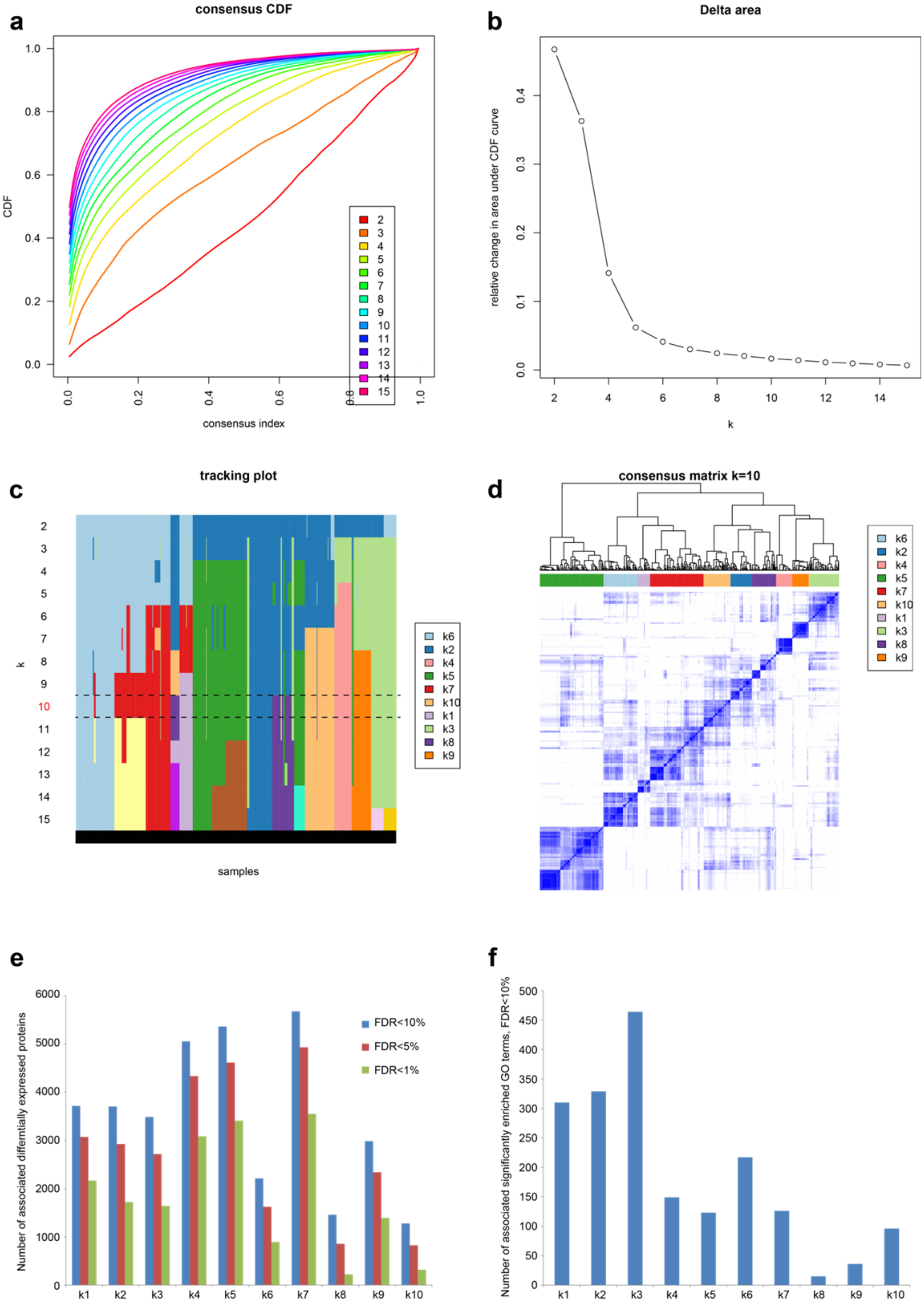
a
consensus CDF
CDF
consensus index
1.0
0.8
0.6
0.4
0.2
0.0
2
3
4
5
6
7
8
9
10
11
12
13
14
15
b
Delta area
relative change in area under CDF curve
k
0.4
0.3
0.2
0.1
0.0
2
4
6
8
10
12
14
c
tracking plot
k
samples
k6
k2
k4
k5
k7
k10
k1
k3
k8
k9
d
consensus matrix k=10
k6
k2
k4
k5
k7
k10
k1
k3
k8
k9
e
Number of associated differentially expressed proteins
FDR<10%
FDR<5%
FDR<1%
6000
5000
4000
3000
2000
1000
0
k1
k2
k3
k4
k5
k6
k7
k8
k9
k10
f
Number of associated significantly enriched GO terms, FDR<10%
500
450
400
350
300
250
200
150
100
50
0
k1
k2
k3
k4
k5
k6
k7
k8
k9
k10

Supplementary Figure 2, related to Figure 2. Derivation of *de novo* pan-cancer molecular subtypes as defined by mass-spectrometry-based proteomics. (a) Graphic shows the cumulative distribution functions (CDFs) of the consensus matrix for each k (indicated by colors) in the clustering results, estimated by a histogram of 100 bins. This graphic was used as a guide to determine at what number of clusters, k, the CDF reaches an approximate maximum. **(b)** Delta area plot graphic showing the relative change in area under the CDF curve comparing k and k − 1. For k = 2, there is no k -1, so the total area under the curve rather than the relative increase is plotted. This graphic allows one to determine the relative increase in consensus and determine k at which there is no appreciable increase. **(c)** Tracking plot graphic shows the cluster assignment of items (columns) for each k (rows) by color, from k=2 to k=15. The colors correspond to the colors of the consensus matrix class assignments. This plot provides a view of item cluster membership across different k and enables one to track the history of clusters relative to earlier clusters. The k7 subtype (stroma-related but distinct from k6) appears at the k=6 solution and up. The k10 subtype (endoplasmic reticulum-related) appears at the k=7 solution and up. The k9 solution (hemoglobin complex- and renal cancer-related) appears at the k=8 solution and up. The k1 subtype (metabolism-related and analogous to the "c1" mRNA-based subtype from ref[1]) appears at the k=9 solution and up. The k8 subtype (Golgi apparatus-related) appears at the k=10 solution and up. **(d)** Consensus matrix at k=10, representing pairwise consensus values, the proportion that two profiles occupied the same cluster out of the number of times they occurred in the same subsample. The graphical display would indicate a reasonable cluster number and membership at k=10. (The numbering of clustering here would be different from the k1 through k10 numbering convention used elsewhere.) **(e)** For each k=10 subtype, numbers of significantly differentially expressed genes, comparing tumors of the given subtype versus the rest of the tumors. FDR, False Discovery Rate. **(f)** For each k=10 subtype, numbers of significantly enriched GO terms (FDR<10%). For the top 100 over-expressed proteins associated with each subtype (from Figure 2b), represented categories by GO were assessed by one-sided Fisher's exact test. Beyond the use of raw analytics, our proteome-based pan-cancer subtypes were found to show good concordance with results of external and independent datasets (main Figure 3) and would show evidence of distinctive biology on the basis of pathway and integrative analyses. While additional molecular subtypes could be defined arbitrarily (e.g. by increasing the ConsensusClusterPlus k, parts c and d), these would also need to have some unique biology associations for them to be relevant. Future studies involving larger numbers of cancer cases or different cancer types may identify additional pan-cancer subtypes.

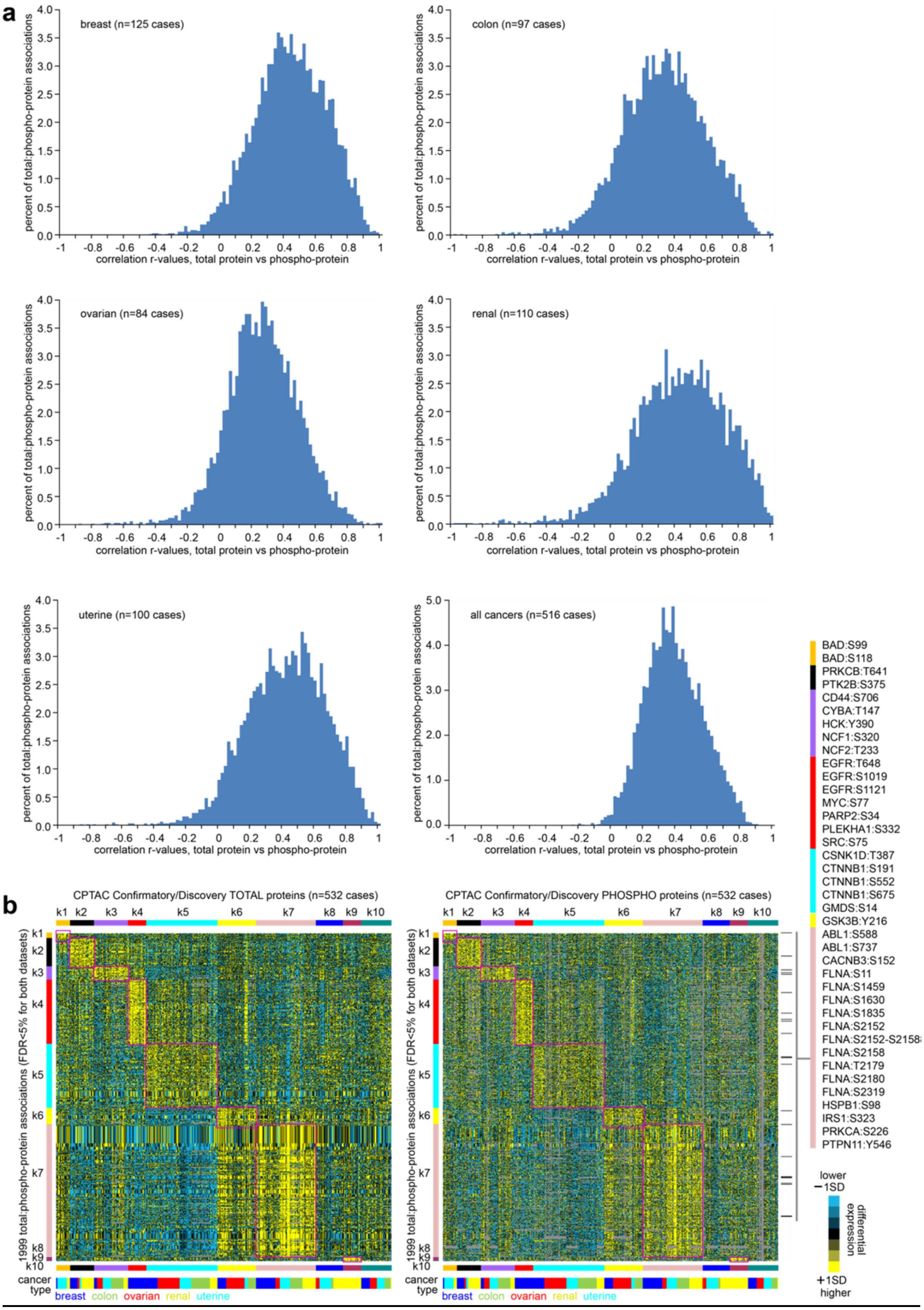
a
breast (n=125 cases)
colon (n=97 cases)
ovarian (n=84 cases)
renal (n=110 cases)
uterine (n=100 cases)
all cancers (n=516 cases)
percent of total:phospho-protein associations
correlation r-values, total protein vs phospho-protein
b
CPTAC Confirmatory/Discovery TOTAL proteins (n=532 cases)
CPTAC Confirmatory/Discovery PHOSPHO proteins (n=532 cases)
k1 k2 k3 k4 k5 k6 k7 k8 k9 k10
1999 total:phospho-protein associations (FDR<5% for both datasets)
cancer type
breast colon ovarian renal uterine
BAD:S99
BAD:S118
PRKCB:T641
PTK2B:S375
CD44:S706
CYBA:T147
HCK:Y390
NCF1:S320
NCF2:T233
EGFR:T648
EGFR:S1019
EGFR:S1121
MYC:S77
PARP2:S34
PLEKHA1:S332
SRC:S75
CSNK1D:T387
CTNNB1:S191
CTNNB1:S552
CTNNB1:S675
GMDS:S14
GSK3B:Y216
ABL1:S588
ABL1:S737
CACNB3:S152
FLNA:S11
FLNA:S1459
FLNA:S1630
FLNA:S1835
FLNA:S2152
FLNA:S2152-S2158
FLNA:S2158
FLNA:T2179
FLNA:S2180
FLNA:S2319
HSPB1:S98
IRS1:S323
PRKCA:S226
PTPN11:Y546
lower
−1SD
differential expression
+1SD
higher

Supplementary Figure 3, related to Figure 2. Correlations between total and phosphorylated protein forms. (a) Histograms of Pearson's correlation r-values between total and phosphorylated forms for the same protein. Correlations assessed separately across profiles in CPTAC Breast Confirmatory proteomic dataset, CPTAC Colon Confirmatory proteomic dataset, CPTAC Ovarian Confirmatory proteomic dataset, CPTAC Renal Discovery proteomic dataset, CPTAC Uterine Discovery proteomic dataset, and combined CPTAC Confirmatory/Discovery pan-cancer proteomic dataset (with expression values normalized within cancer type). As is evident here, phospho-proteins tended to positively correlate with expression of the total protein, though with correlation r-values much less than 1 for most proteins. **(b)** For each proteome-based subtype, the top over-expressed proteins were defined, in which both a phosphorylated form and its corresponding total form were each significantly high (FDR<5%) in the given subtype versus the rest of the tumors. Differential expression patterns (values normalized within each main cancer type; SD, standard deviation from the median) are represented for these protein features, with phosphorylated protein forms ordered side-by-side with the corresponding total protein form. Proteins individually listed have GO annotation "cell surface receptor signaling pathway" and an association in DrugBank[3] database.

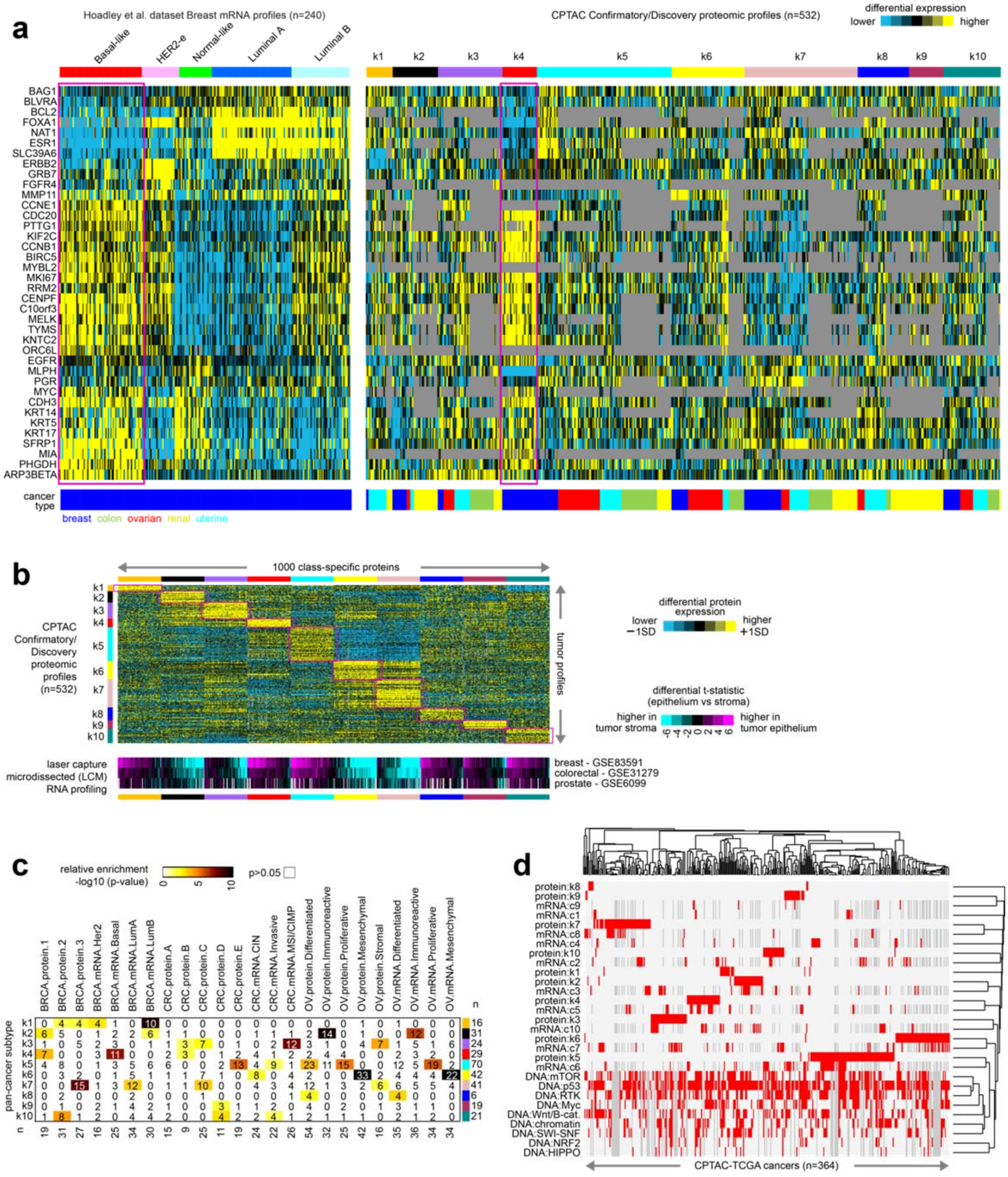

Supplementary Figure 4, related to Figure 2. Comparisons of proteome-based subtypes with molecular subtypes defined using external datasets and previous studies. (a) Proteome-based k4 subtype aligns with basal-like breast cancer. Expression patterns of the PAM50 gene set (38 genes represented in CPTAC datasets) in the mRNA profile dataset from Hoadley et al.[4] and CPTAC Confirmatory/Discovery proteomic dataset are shown. Hoadley basal-like breast cancers and CPTAC k4 tumors

are highlighted. **(b)** For the set of 1000 proteins found to best distinguish between the ten proteome-based subtypes (proteins from Figure 2b, top 100 over-expressed proteins for each of the 10 subtypes as indicated), the corresponding differential mRNA patterns comparing tumor epithelium versus tumor stroma (based on public GEO datasets of previous studies utilizing laser capture microdissection, or LCM[5-7]) are represented. For CPTAC dataset, differential expression values are normalized within each main cancer type; SD, standard deviation from the median. Results indicate that proteins most highly over-expressed in k2, k3, k6, and k7 subtypes tend to represent components of the tumor stroma, while proteins most highly over-expressed in the other subtypes tend to represent the tumor epithelium. **(c)** Significance of overlap between the proteome-based pan-cancer subtype assignments made for TCGA cases in the present study (rows), with molecular-based subtype assignments (columns) made previously for a subset of cases in CPTAC- or TCGA-led studies[8-13]. P-values by one-sided Fisher's exact test. Assignments of proteome-based subtype to TCGA-CPTAC cases are represented in main Figure 3a. Overall, the proteome-based pan-cancer molecular subtypes show significant concordances with other molecular subtype designations. The two immune-related subtypes appear distinct from each other in terms of associations with the previous subtypes (e.g. k2 but not k3 associating with Ovarian Immunoreactive subtype), and the two stroma-related subtypes also appear distinct from each other in terms of associations with the previous subtypes (e.g. k6 but not k7 associating with Ovarian Mesenchymal subtype, and k7 associating the Ovarian Stromal subtype). **(d)** Cluster-of-clusters analysis (COCA)[14], as a first attempt to integrate subtype classifications of the 364 TCGA-CPTAC cases, at the levels of proteome (present study, assignments of proteome-based subtype to TCGA-CPTAC cases represented in main Figure 3a), transcriptome (from ref[1]), and DNA-level pathway alterations (curated for TCGA cases in ref[1]). In the clustered data matrix, red denotes membership for the given cancer case in the given subtype. We see here that DNA-based classifications cluster separately from the proteome-based on transcriptome-based classifications, and that there is some observed overlap between the proteome-based and transcriptome-based classifications (e.g. mRNA.c5/protein.k4, mRNA.c6/protein.k5, mRNA.c7/protein.k6, mRNA.c8/protein.k7).

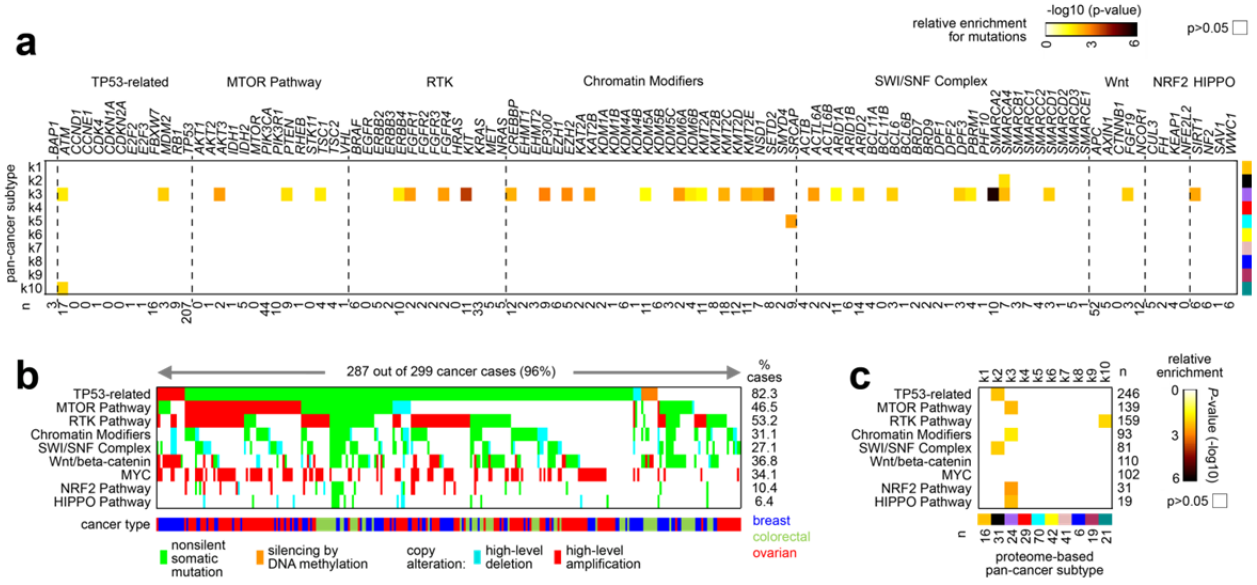

Supplementary Figure 5, related to Figure 3. Somatic mutations and associated pathways across proteome-based pan-cancer subtypes in CPTAC-TCGA cohort. (a) For each proteome-based pan-cancer subtype, significances of enrichment (one-sided Fisher's exact test) of mutation events for each gene within the given subtype versus the rest of the tumors. **(b)** Pathway-centric view of nonsilent gene mutations and copy alterations in CPTAC-TCGA cohort (n=299 cancer cases with available exome sequencing data). "High-level" deletion and "high-level" amplification respectively approximate total copy loss and copy levels more than 2X greater than that of wild-type (based on GISTIC[15] thresholded values). See part a for the genes associated with each pathway. DNA-level pathway alterations previously curated for TCGA cases in ref[1]. **(c)** By pan-cancer subtype, significances of enrichment (one-sided Fisher's exact test) of gene alteration events for each pathway within any particular subtype versus the rest of the cases. The above results indicate that while a number of pathway-level or individual gene-level DNA alterations surveyed were moderately represented within specific pan-cancer subtypes, no strong connections between tumor oncogenotypes and proteome-based subtypes were evident.

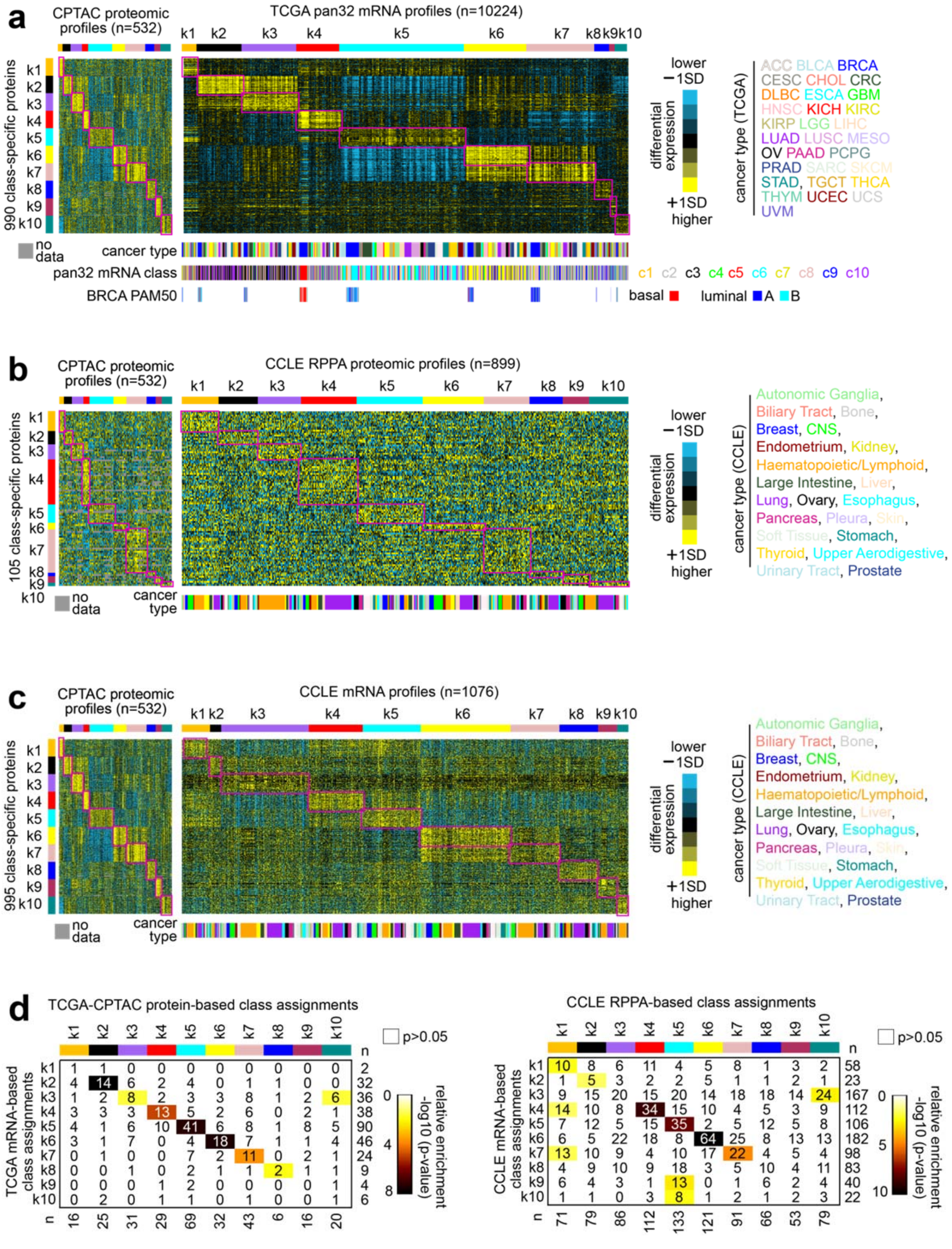
a
CPTAC proteomic profiles (n=532)
TCGA pan32 mRNA profiles (n=10224)
k1 k2 k3 k4 k5 k6 k7 k8 k9 k10
990 class-specific proteins
lower −1SD
differential expression
+1SD higher
cancer type (TCGA)
ACC BLCA BRCA
CESC CHOL CRC
DLBC ESCA GBM
HNSC KICH KIRC
KIRP LGG LIHC
LUAD LUSC MESO
OV PAAD PCPG
PRAD SARC SKCM
STAD, TGCT THCA
THYM UCEC UCS
UVM
no data
cancer type
pan32 mRNA class
c1 c2 c3 c4 c5 c6 c7 c8 c9 c10
BRCA PAM50
basal
luminal A B
b
CPTAC proteomic profiles (n=532)
CCLE RPPA proteomic profiles (n=899)
k1 k2 k3 k4 k5 k6 k7 k8 k9 k10
105 class-specific proteins
lower −1SD
differential expression
+1SD higher
cancer type (CCLE)
Autonomic Ganglia,
Biliary Tract, Bone,
Breast, CNS,
Endometrium, Kidney,
Haematopoietic/Lymphoid,
Large Intestine, Liver,
Lung, Ovary, Esophagus,
Pancreas, Pleura, Skin,
Soft Tissue, Stomach,
Thyroid, Upper Aerodigestive,
Urinary Tract, Prostate
no data
cancer type
c
CPTAC proteomic profiles (n=532)
CCLE mRNA profiles (n=1076)
k1 k2 k3 k4 k5 k6 k7 k8 k9 k10
995 class-specific proteins
lower −1SD
differential expression
+1SD higher
cancer type (CCLE)
Autonomic Ganglia,
Biliary Tract, Bone,
Breast, CNS,
Endometrium, Kidney,
Haematopoietic/Lymphoid,
Large Intestine, Liver,
Lung, Ovary, Esophagus,
Pancreas, Pleura, Skin,
Soft Tissue, Stomach,
Thyroid, Upper Aerodigestive,
Urinary Tract, Prostate
no data
cancer type
d
TCGA-CPTAC protein-based class assignments
TCGA mRNA-based class assignments
k1 k2 k3 k4 k5 k6 k7 k8 k9 k10 n
k1 1 1 0 0 0 0 0 0 0 0 2
k2 4 14 6 2 4 0 1 1 0 0 32
k3 1 2 8 2 3 3 8 1 2 6 36
k4 3 3 3 13 5 2 6 0 2 1 38
k5 4 1 6 10 41 6 8 1 8 5 90
k6 3 1 7 0 4 18 7 1 1 4 46
k7 0 1 0 0 7 2 11 0 2 1 24
k8 0 0 1 0 2 1 1 2 1 1 9
k9 0 0 0 1 2 0 0 0 0 1 4
k10 0 2 0 1 1 0 1 0 0 1 6
n 16 25 31 29 69 32 43 6 16 20
p>0.05
relative enrichment -log10 (p-value)
0 4 8
CCLE RPPA-based class assignments
CCLE mRNA-based class assignments
k1 k2 k3 k4 k5 k6 k7 k8 k9 k10 n
k1 10 8 6 11 4 5 8 1 3 2 58
k2 1 5 3 2 2 5 1 1 2 1 23
k3 9 15 20 15 20 14 18 18 14 24 167
k4 14 10 8 34 15 10 4 5 3 9 112
k5 7 12 5 15 35 2 5 12 5 8 106
k6 6 5 22 18 8 64 25 8 13 13 182
k7 13 10 9 4 10 17 22 4 5 4 98
k8 4 9 10 9 18 3 5 10 4 11 83
k9 6 4 3 1 13 0 1 6 2 4 40
k10 1 1 0 3 8 1 2 1 2 3 22
n 71 79 86 112 133 121 91 66 53 79
p>0.05
relative enrichment -log10 (p-value)
0 5 10

Supplementary Figure 6, related to Figure 3. Observation of patterns associated with CPTAC pan-cancer proteome-based subtypes in additional multi-cancer transcriptome and proteome datasets. (a) The 10224 TCGA pan32 cases with transcriptome data were classified according to proteome-based pan-cancer subtype as originally defined using CPTAC Confirmatory/Discovery cohort. Expression patterns for the top set of 990 proteins distinguishing between the ten subtypes (from Figure 2a, based on available data, mapping between protein and mRNA) are shown for both CPTAC Confirmatory/Discovery proteomic and TCGA pan32 datasets (values normalized within each main cancer type; SD, standard deviation from the median). Gene patterns in the TCGA sample profiles sharing similarity with a subtype-specific signature pattern are highlighted. **(b)** A set of 899 human cancer cell lines from the Cancer Cell Line Encyclopedia (CCLE) with Reverse-Phase Protein Array (RPPA) data were classified according to proteome-based pan-cancer subtype. Expression patterns for a top set of 105 proteins distinguishing between the ten subtypes (Methods, based on available data) are shown for both CPTAC Confirmatory/Discovery and CCLE RPPA proteomics datasets. Gene patterns in the CCLE sample profiles sharing similarity with a subtype-specific signature pattern are highlighted. **(c)** A set of 1076 human cancer cell lines from the CCLE with transcriptome data were classified according to proteome-based pan-cancer subtype. Expression patterns for a top set of 105 proteins distinguishing between the ten subtypes (from Figure 2a, based on available data, mapping between protein and mRNA) are shown for both CPTAC Confirmatory/Discovery and CCLE transcriptomic datasets. Gene patterns in the CCLE sample profiles sharing similarity with subtype-specific signature pattern are highlighted. **(d)** *Left:* Significances of overlap between the proteome-based subtype assignments made for the CPTAC-TCGA dataset (columns), with subtype assignments for the transcriptome profiles in TCGA pan32 cohort (rows, mapping the CPTAC protein expression patterns to TCGA mRNA patterns), based on the 287 cases represented in both datasets. *Right:* Significances of overlap between the RPPA-based subtype assignments made for the CCLE dataset (columns), with subtype assignments for the transcriptome profiles in CCLE (rows), based on the 891 cell lines represented in both datasets. P-values by one-sided Fisher's exact test. Several proteome-based tumor subtypes appear manifested *in vitro* in cancer cell lines, though the associated differential patterns do not appear as strong as for the tumor data.

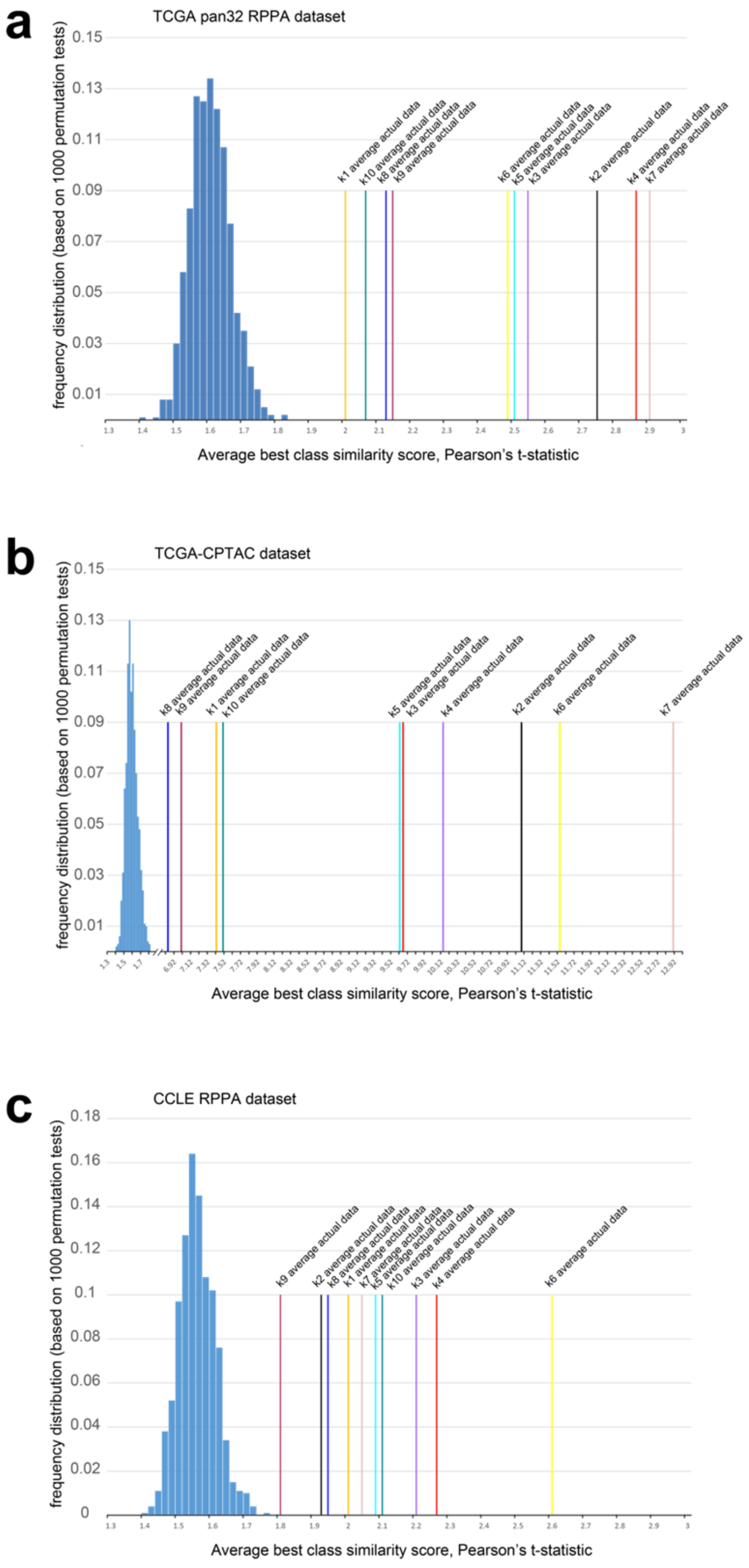
a
TCGA pan32 RPPA dataset
frequency distribution (based on 1000 permutation tests)
Average best class similarity score, Pearson's t-statistic
k1 average actual data
k10 average actual data
k8 average actual data
k9 average actual data
k6 average actual data
k5 average actual data
k3 average actual data
k2 average actual data
k4 average actual data
k7 average actual data
b
TCGA-CPTAC dataset
frequency distribution (based on 1000 permutation tests)
Average best class similarity score, Pearson's t-statistic
k8 average actual data
k9 average actual data
k1 average actual data
k10 average actual data
k5 average actual data
k3 average actual data
k4 average actual data
k2 average actual data
k6 average actual data
k7 average actual data
c
CCLE RPPA dataset
frequency distribution (based on 1000 permutation tests)
Average best class similarity score, Pearson's t-statistic
k9 average actual data
k2 average actual data
k8 average actual data
k1 average actual data
k7 average actual data
k5 average actual data
k10 average actual data
k3 average actual data
k4 average actual data
k6 average actual data

Supplementary Figure 7, related to Figure 3. Assessment of the overall strength of the correlations behind the CPTAC proteome-based subtype assignments made to external datasets. (a) Histogram of results from 1000 random permutations of the subtype assignments for TCGA pan32 RPPA dataset; in each permutation test, the gene ordering of the TCGA RPPA dataset was made random relative to CPTAC subtype classifier, and subtype assignments were made using the “best fit” class with the highest correlation. The distribution of the average best fit subtype correlations from each of the permuted datasets are shown (representing 1000 best subtype similarity scores), along with the average best fit correlations for each of the ten pan-cancer classes in the actual, non-permuted datasets. **(b)** Similar to part a, but for CPTAC-TCGA dataset. **(c)** Similar to part a, but for the CCLE RPPA dataset.

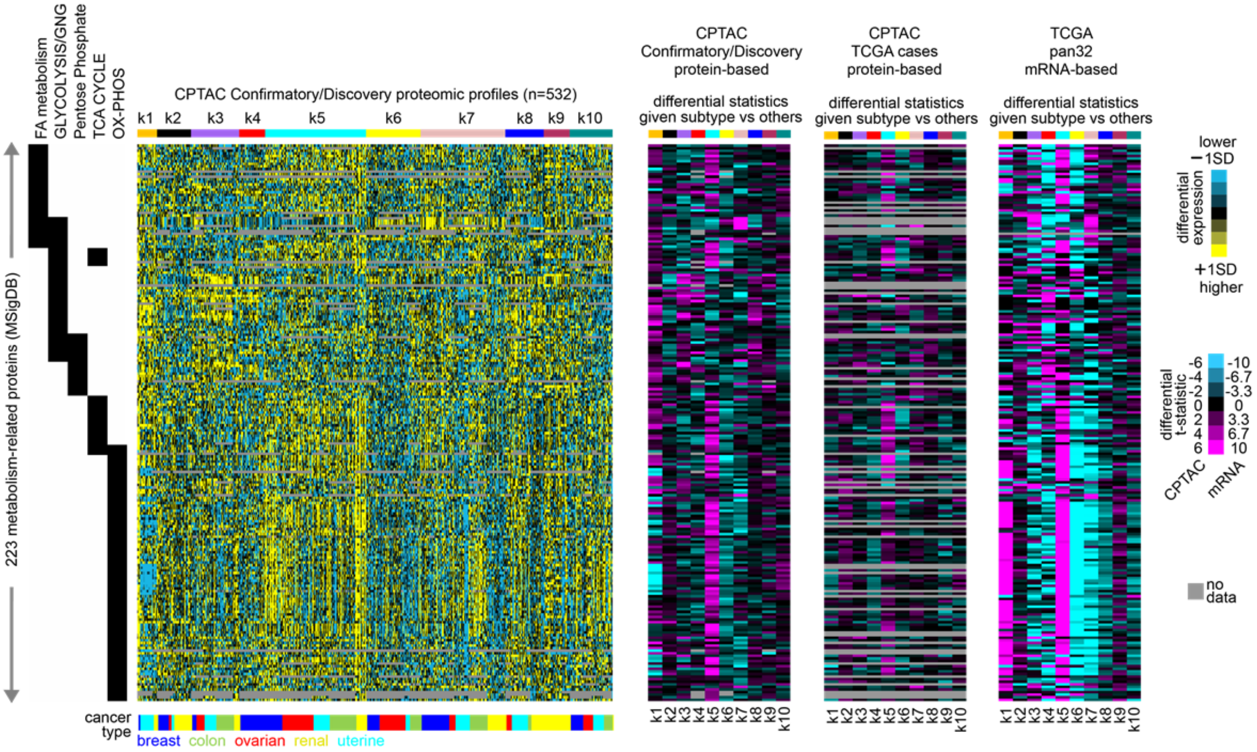

Supplementary Figure 8, related to Figure 4. Protein-level differences involving gene signatures of altered pathways. For CPTAC Confirmatory/Discovery proteomic dataset, protein-level patterns involved pathway-associated gene signatures (using values normalized within each cancer type; SD, standard deviation from the median) are represented. Purple-cyan heat maps denote t-statistics for comparing the given subtype versus the other tumors, for each of the following datasets: CPTAC Confirmatory/Discovery proteomic, CPTAC-TCGA proteomic, and TCGA pan32 mRNA.

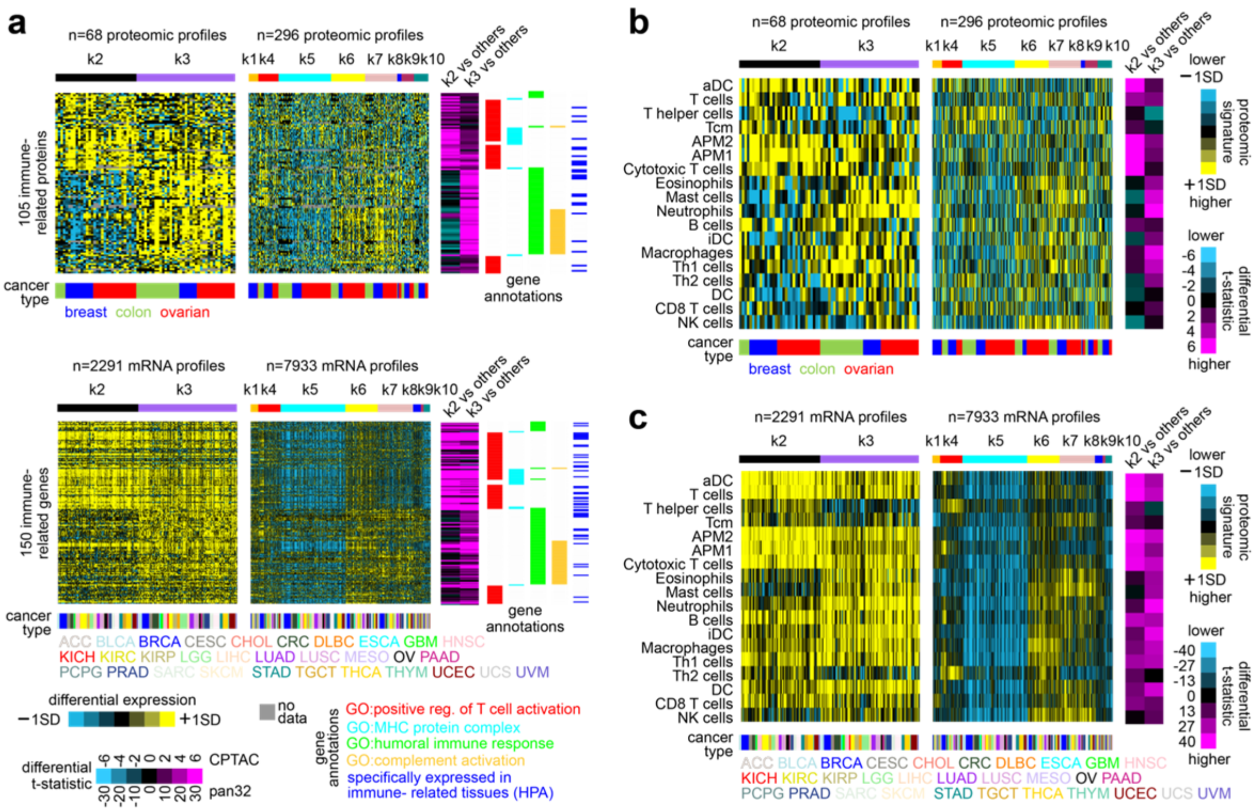

Supplementary Figure 9, related to Figure 5. (a) For the set of 162 immune-related proteins featured in Figure 5a, the differential protein expression patterns (expression values normalized within cancer type; SD, standard deviation from the median) were examined in both the CPTAC-TCGA proteomic dataset (top) and the TCGA pan32 mRNA dataset (bottom), with profiles ordered by subtype and using genes or proteins with available data. Purple-cyan heat map denotes t-statistics for comparing the given subtype versus the other tumors. **(b)** Heat maps of gene expression-based signatures[16] of immune cell infiltrates, across CPTAC-TCGA proteomic profiles, ordered by subtype (expression values normalized within cancer type; SD, standard deviation from the median). Purple-cyan heat map denotes t-statistics for comparing the given subtype versus the other tumors. **(c)** Similar to part b, but for TCGA pan32 mRNA dataset.

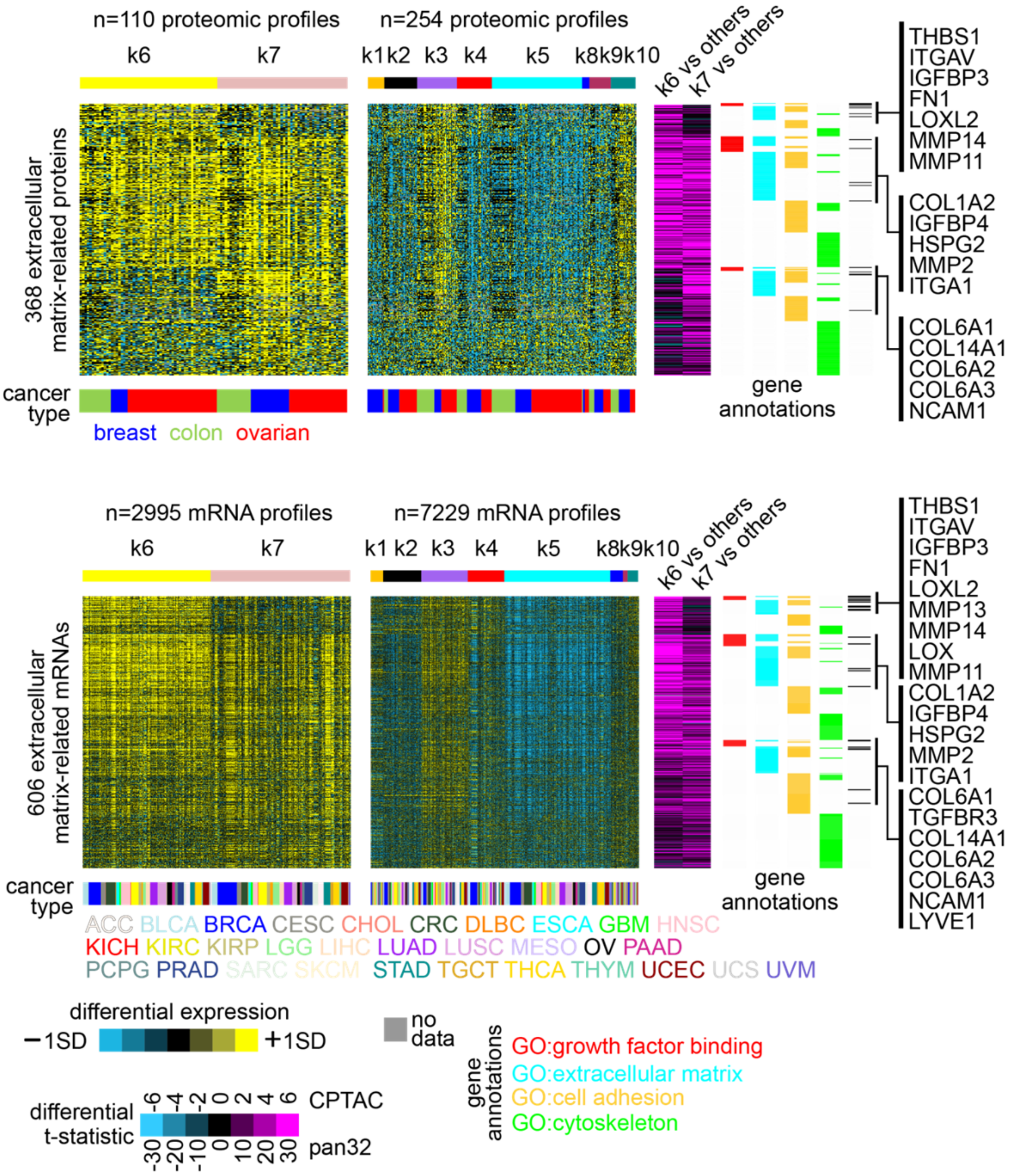

Supplementary Figure 10, related to Figure 6. For the set of 606 extracellular matrix-related proteins featured in Figure 6a, the differential protein expression patterns (expression values normalized within cancer type; SD, standard deviation from the median) were examined in both the CPTAC-TCGA proteomic dataset (top) and the TCGA pan32 mRNA dataset (bottom), with profiles ordered by subtype and using genes

or proteins with available data. Purple-cyan heat map denotes t-statistics for comparing the given subtype versus the other tumors.

Supplementary References

1. Chen, F.*, et al.* Pan-cancer molecular classes transcending tumor lineage across 32 cancer types, multiple data platforms, and over 10,000 cases. *Clin Cancer Res.* **24**, 2182-2193 (2018).
2. Ashburner, M.*, et al.* Gene ontology: tool for the unification of biology. The Gene Ontology Consortium. *Nature genetics* **25**, 25-29 (2000).
3. Law, V.*, et al.* DrugBank 4.0: shedding new light on drug metabolism. *Nucleic Acids Res* **42**, D1091-1097 (2014).
4. Hoadley, K.*, et al.* EGFR associated expression profiles vary with breast tumor subtype. *BMC Genomics* **8** (2007).
5. Tomlins, S.*, et al.* Integrative molecular concept modeling of prostate cancer progression. *Nature genetics* **39**, 41-51 (2007).
6. Liu, H.*, et al.* Discovery of Stromal Regulatory Networks that Suppress Ras-Sensitized Epithelial Cell Proliferation. *Dev Cell* **41**, 392-407 (2017).
7. Abba, M.*, et al.* Look who's talking: deregulated signaling in colorectal cancer. *Cancer Genomics Proteomics* **9**, 15-25 (2012).
8. Zhang, B.*, et al.* Proteogenomic characterization of human colon and rectal cancer. *Nature* **513**, 382-387 (2014).
9. Mertins, P.*, et al.* Proteogenomics connects somatic mutations to signalling in breast cancer. *Nature* **534**, 55-62 (2016).
10. Zhang, H.*, et al.* Integrated Proteogenomic Characterization of Human High-Grade Serous Ovarian Cancer. *Cell* **166**, 755-765 (2016).
11. The_Cancer_Genome_Atlas_Network. Comprehensive molecular portraits of human breast tumours. *Nature* **490**, 61-70 (2012).
12. Cancer_Genome_Atlas_Network. Comprehensive molecular characterization of human colon and rectal cancer. *Nature* **487**, 330-337 (2012).
13. Cancer_Genome_Atlas_Research_Network. Integrated genomic analyses of ovarian carcinoma. *Nature* **474**, 609-615 (2011).
14. Hoadley, K.*, et al.* Multiplatform Analysis of 12 Cancer Types Reveals Molecular Classification within and across Tissues of Origin. *Cell* **158**, 929-944 (2014).
15. Mermel, C.H.*, et al.* GISTIC2.0 facilitates sensitive and confident localization of the targets of focal somatic copy-number alteration in human cancers. *Genome biology* **12**, R41 (2011).
16. Bindea, G.*, et al.* Spatiotemporal dynamics of intratumoral immune cells reveal the immune landscape in human cancer. *Immunity* **39**, 782-795 (2013).
