# Supplementary material for: Pan-cancer molecular subtypes revealed by mass-spectrometry-based proteomic characterization of more than 500 human cancers
Source: Nat Commun. 2019 Dec 12;10:5679. doi: 10.1038/s41467-019-13528-0 (PMC6908580; doi:10.1038/s41467-019-13528-0)
Supplement: Supplementary file 3 — Description of Additional Supplementary Files [file 41467_2019_13528_MOESM3_ESM.pdf]

Description of Additional Supplementary Files

File Name: Supplementary Data 1
Description: Patient-level subtyping and molecular features. Provided as an Excel file. Results are provided for the CPTAC Confirmation/Discovery cohort, the CPTAC-TCGA cohort, the TCGA pan32 mRNA/RPPA cohort, and the Cancer Cell Line Encyclopedia (CCLE). Results include assigned subtypes (e.g. CPTAC proteome-based subtype and TCGA mRNA-based subtype), pathway signature scoring, and immune signature scoring.

File Name: Supplementary Data 2
Description: Protein-level correlations with proteome-based subtype. Provided as an Excel file. Results are provided for the CPTAC Confirmation/Discovery total protein and phospho-protein datasets, as well as for the CPTAC-TCGA total protein dataset. For data tab with CPTAC Confirmation/Discovery total protein features, columns in Excel provide protein-level correlations with each proteome-based subtype (columns F-AI, including t-statistics and associated p-values and false discovery rates), membership in selected GO term categories (focusing on GO terms used for main Figures 5-7, columns AJ-AV), protein-level correlations with each mRNA-based subtype (c1-c10, columns AW-BF), membership in selected genes lists including set of top 1000 differential proteins by subtype from Figure 2B (top 100 over-expressed for each subtype k1-k10, columns BG-BI), antibody availability for RPPA or IHC studies (BJ-BK), and tumor epithelium vs tumor stroma comparisons at the mRNA level (based on public GEO datasets of studies utilizing LCM, columns BL-BN).

File Name: Supplementary Data 3
Description: Proteome-based subtype classifiers. Provided as an Excel file. Results include the top differential expression patterns (values normalized within each main cancer type) for the set of 1000 total proteins and the set of 500 phosphoproteins represented in figure 2b. The values for the top 1000 total proteins were used as the basis of the classifier applied to CPTAC-TCGA and TCGA pan32 mRNA datasets. The set of protein features used to classify the TCGA-RPPA dataset is also provided. In a separate tab, "classify CPTAC-TCGA by k1-k10," calculations are provided in Excel, by which the CPTAC-TCGA proteomic profiles were classified according to proteome-based pan-cancer subtype (assigned subtype is computed in row 1, referring to calculations in rows 1005-1016, results used for Figure 3a).

File Name: Supplementary Data 4
Description: Gene Ontology (GO) annotation term associations for the top proteins over-expressed within each of the proteome-based subtypes. Provided as an Excel file. For each subtype, the top 100 over-expressed proteins (from Figure 2b) were searched for GO term enrichment.

File Name: Supplementary Data 5
Description: Protein interaction networks associated with k6, k7, k8, k9, and k10 subtypes. Provided as an Excel file.

File Name: Supplementary Data 6
Description: R source code written for this study. R code was used to define proteome-based subtypes (by ConsensusClusterPlus R package), using a data expression matrix of the top 2000 most variable proteins from the CPTAC Confirmatory/Discovery total protein dataset; code and matrix file

are provided in the “subtype_discovery” zip file. R code was also used to generate 1000 random permutations of the subtype assignments for the datasets external to CPTAC Confirmatory/Discovery (whereby in each permutation test, the gene ordering of the external dataset was made random relative to CPTAC subtype classifier, and subtype assignments were made using the “best fit” class with the highest correlation, results presented in Supplementary Figure 7); code and data matrix files are provided in the “permutation_testing” zip file.
