# Supplementary material for: Pan-cancer molecular subtypes revealed by mass-spectrometry-based proteomic characterization of more than 500 human cancers
Source: Nat Commun. 2019 Dec 12;10:5679. doi: 10.1038/s41467-019-13528-0 (PMC6908580; doi:10.1038/s41467-019-13528-0)
Supplement: Supplementary file 10 — Reporting Summary [file 41467_2019_13528_MOESM10_ESM.pdf]

nature research

Corresponding author(s): Chad Creighton

Last updated by author(s): 2019-10-2

Reporting Summary

Nature Research wishes to improve the reproducibility of the work that we publish. This form provides structure for consistency and transparency in reporting. For further information on Nature Research policies, see Authors & Referees and the Editorial Policy Checklist.

Statistics

For all statistical analyses, confirm that the following items are present in the figure legend, table legend, main text, or Methods section.

n/a	Confirmed	
☐	☒	The exact sample size (*n*) for each experimental group/condition, given as a discrete number and unit of measurement
☐	☒	A statement on whether measurements were taken from distinct samples or whether the same sample was measured repeatedly
☐	☒	The statistical test(s) used AND whether they are one- or two-sided<br>*Only common tests should be described solely by name; describe more complex techniques in the Methods section.*
☐	☒	A description of all covariates tested
☐	☒	A description of any assumptions or corrections, such as tests of normality and adjustment for multiple comparisons
☐	☒	A full description of the statistical parameters including central tendency (e.g. means) or other basic estimates (e.g. regression coefficient) AND variation (e.g. standard deviation) or associated estimates of uncertainty (e.g. confidence intervals)
☐	☒	For null hypothesis testing, the test statistic (e.g. *F*, *t*, *r*) with confidence intervals, effect sizes, degrees of freedom and *P* value noted<br>*Give P values as exact values whenever suitable.*
☐	☒	For Bayesian analysis, information on the choice of priors and Markov chain Monte Carlo settings
☐	☒	For hierarchical and complex designs, identification of the appropriate level for tests and full reporting of outcomes
☐	☒	Estimates of effect sizes (e.g. Cohen's *d*, Pearson's *r*), indicating how they were calculated

Our web collection on statistics for biologists contains articles on many of the points above.

Software and code

Policy information about availability of computer code

Data collection	All data used in this study are publicly available. CPTAC data are available through the CPTAC data portal (https://cptac-data-portal.georgetown.edu/cptacPublic/).
Data analysis	R source code written for this study is provided as part of Supplementary Data 6.

For manuscripts utilizing custom algorithms or software that are central to the research but not yet described in published literature, software must be made available to editors/reviewers. We strongly encourage code deposition in a community repository (e.g. GitHub). See the Nature Research guidelines for submitting code & software for further information.

Data

Policy information about availability of data

All manuscripts must include a data availability statement. This statement should provide the following information, where applicable:

- Accession codes, unique identifiers, or web links for publicly available datasets
- A list of figures that have associated raw data
- A description of any restrictions on data availability

All data used in this study are publicly available. CPTAC data are available through the CPTAC data portal (https://cptac-data-portal.georgetown.edu/cptacPublic/). TCGA data are available through the Genome Data Commons (https://gdc.cancer.gov/) and the Broad Institute's Firehose data portal (https://gdac.broadinstitute.org). Cancer Cell Line Encyclopedia (CCLE) datasets are available from http://www.broadinstitute.org/ccle. All publication moratoriums as set by the respective consortiums for utilizing these data were respectfully followed, with the CPTAC Breast Confirmatory dataset being the last to have its moratorium lifted as of July 15, 2019.

Field-specific reporting

Please select the one below that is the best fit for your research. If you are not sure, read the appropriate sections before making your selection.

☒ Life sciences ☐ Behavioural & social sciences ☐ Ecological, evolutionary & environmental sciences

For a reference copy of the document with all sections, see nature.com/documents/nr-reporting-summary-flat.pdf

Life sciences study design

All studies must disclose on these points even when the disclosure is negative.

Sample size	The primary focus of this study was on a set of mass-spectrometry-based proteomic profiles from the CPTAC "Confirmatory/Discovery" cohort, of 532 cancer cases (cases not represented in TCGA), comprised of 125 breast cancer cases, 97 colon, 100 ovarian, 110 renal (clear cell renal cell carcinoma), and 100 uterine (Supplementary Data S1). This CPTAC Confirmatory/Discovery dataset was used to define proteome-based pan-cancer subtypes cutting across tissue-specific differences between the cancer types. Additional proteome and transcriptome datasets of TCGA cancer cases, which shared no cases with the CPTAC Confirmatory/Discovery cohort, were used to independently confirm the observations initially made using CTPAC Confirmatory/Discovery dataset (Figure 1a). In the "CPTAC-TCGA" proteome dataset, a subset of 364 cases from TCGA—involving breast, colorectal, and ovarian cancers (105, 90, and 169 cases, respectively, Supplementary Data S1)—was also profiled by mass-spectrometry-based proteomics. TCGA RPPA dataset represented 7663 TCGA cases and 31 cancer types profiled for a focused panel of proteins (involving 211 protein features6 or 169 unique genes) by antibody-based Reverse-Phase Protein Array (RPPA) platform. TCGA "pan32" mRNA dataset featured 10224 cases and 32 cancer types with RNA-sequencing data.
Data exclusions	No data were excluded from the analysis.
Replication	Not applicable, no experimental data
Randomization	Not applicable, no experimental data
Blinding	Blinding not relevant, as samples were not allocated to experimental groups.

Reporting for specific materials, systems and methods

We require information from authors about some types of materials, experimental systems and methods used in many studies. Here, indicate whether each material, system or method listed is relevant to your study. If you are not sure if a list item applies to your research, read the appropriate section before selecting a response.

Materials & experimental systems

n/a	Involved in the study
☒	☐ Antibodies
☒	☐ Eukaryotic cell lines
☒	☐ Palaeontology
☒	☐ Animals and other organisms
☒	☐ Human research participants
☒	☐ Clinical data

Methods

n/a	Involved in the study
☒	☐ ChIP-seq
☒	☐ Flow cytometry
☒	☐ MRI-based neuroimaging
